# Design and Fabrication of Bilayer Hydrogel System with Self-Healing and Detachment Properties Achieved by Near-Infrared Irradiation

**DOI:** 10.3390/polym9060237

**Published:** 2017-06-20

**Authors:** Qian Zhao, Wenhua Hou, Yunhong Liang, Zhihui Zhang, Luquan Ren

**Affiliations:** The Key Laboratory of Bionic Engineering, Ministry of Education, Jilin University, Changchun 130025, China; qianzhao16@mails.jlu.edu.cn (Q.Z.); houwh16@mails.jlu.edu.cn (W.H.); lqren@jlu.edu.cn (L.R.)

**Keywords:** bilayer hydrogel system, self-healing degree, detachment rate, photothermal energy transformation efficiency

## Abstract

A novel kind of graphene oxide (GO)-containing bilayer hydrogel system with excellent self-healing and detachment properties stimulated by near-infrared irradiation is successively fabricated via a two-step in situ free radical polymerization. In addition to high mechanical strength, as components of a bilayer hydrogel system, a poly *N*,*N*-dimethylacrylamide (PDMAA) layer with 3 mg/mL GO and a poly *N*-isopropylacrylamide (PNIPAm) layer with 3 mg/mL GO exhibits firm interface bonding. GO in a PDMAA layer transforms under a near-infrared laser into heat, which promotes mutual diffusion of hydrogen bonds and realizes a self-healing property. The irradiation of near infrared laser results in the temperature of PNIPAm layer being higher than the volume phase transition temperature, reducing the corresponding biological viscidity and achieving detachment property. The increase of GO content enhances the self-healing degree and detachment rate. The bilayer hydrogel system fabricated via mold design combines characteristics of PDMAA layer and PNIPAm layer, which can be treated as materials for medical dressings, soft actuators, and robots.

## 1. Introduction

Due to the chemical or physical cross-linked three-dimensional network and large amounts of water, hydrogels have been widely used in medical science, tissue engineering, soft robots, and soft actuators [1,2,3,4,5]. In order to ensure the application of functions of hydrogels and reduce the manufacturing cost, many studies have been focused on fabricating repairable hydrogels. With the development of smart hydrogels, imitating the living creatures in nature with self-healing properties and fabricating a kind of smart hydrogel which can recover the destroyed part under the stimulations can extend the practical application of self-healable hydrogels. Ceylan and co-workers [6] fabricated a kind of mussel-inspired dynamic cross-linking of a self-healing peptide nanofiber network, which promoted the development of high-performance hydrogels that can remain mechanically stable under abrasive conditions while retaining surface versatility and environmental friendliness. Under the irritation of electricity and electromagnetic waves, Huang et al. [7] fabricated a novel self-healing material with high mechanical properties. Nakahata and co-workers [8] fabricated a kind of redox-responsive self-healing supramolecular hydrogel system via mixing poly(acrylic acid), possessing β-cyclodextrin as a host polymer, with poly(acrylic acid), possessing ferrocene as a guest polymer. In addition to pH, electricity, electromagnetic waves, and redox potential, due to the characteristics of non-contact control, avoiding the restriction of solution, photo-driving self-healing hydrogels has attracted much more attention. Yunseon et al. successfully fabricated a kind of self-healing polymer and composite utilizing the Diels–Alder reaction, which remained stable in extreme environments [9]. After using the thermoresponsive characteristic of graphene oxide, Zhang and co-workers [10] fabricated a kind of poly(*N*,*N*-dimethylacrylamide) hydrogel with enhanced mechanical properties and fast self-healing capability, realized by near-infrared irradiation. Among the previous studies, even though the fabricated hydrogels exhibit high self-healing properties, the corresponding hydrogels show relatively lower biological viscidity in practical applications. Therefore, adding biological viscidity into self-healing hydrogels enhances the corresponding practicability and research significance.

In order to solve the biological viscidity of hydrogels, many studies have been investigated. Gaharwar et al. [11] fabricated a kind of transparent, elastomeric, and tough hydrogel from poly(ethylene glycol) and silicate nanoparticles, with the combined properties of elasticity, stiffness, interconnected network, adhesiveness to surfaces, and bio-adhesion to cells, and provided the inspiration and opportunities to engineer mechanically strong and elastic tissue matrices for orthopedic, craniofacial, and dental applications. Wu and co-workers [12] built a systematic approach to develop robust and adhesive hydrogels by photopolymerizing poly(ethylene glycol)-diacrylate and methoxy-poly(ethylene glycol)-acrylate in the presence of charged silicate nanoparticles, which may aid the development of adhesive tissue engineering matrices, wound dressings, sealants, and the adhesive components of biomedical devices. Via adding α-cyclodextrin into poly(*N*-isopropylacrylamide) hydrogel, Wang et al. [13] improved the stickiness of hydrogels. The produced hydrogel has potential application in adhesive drug delivery systems and wound dressing materials on human skin. Many types of hydrogels with excellent biological viscidity have been investigated, but how to realize the detachment property without damage, especially combined with the self-healing property, has not been studied. Due to the thermoresponsive characteristics, the monomer *N*-isopropylacrylamide has been widely used in poly(*N*-isopropylacrylamide) hydrogels with a specific volume phase transition temperature. Based on the high photothermal energy transformation and absorbance of near-infrared irradiation [14,15,16], graphene oxide changes the thermoresponsive hydrogel to a photoresponsive hydrogel [17,18,19], which has been treated as a material for smart soft robots and actuators based on the phase transition [20,21,22,23]. Therefore, poly(*N*-isopropylacrylamide)-graphene oxide-α-cyclodextrin hydrogel is the best candidate for the fabrication of hydrogels with biological viscidity. Moreover, this type of hydrogel can realize a detachment property via the phase transition under the irradiation of a near-infrared laser. However, because of the differences of the monomers, the self-healing hydrogel and biological viscidity hydrogel cannot use the same monomer. How to combine the two kinds of hydrogels with different monomers and design an effective structure to realize a rapid self-healing degree and detachment property is the key point of our study.

In this paper, after designing and fabricating a set of molds, a novel type of graphene oxide-containing bilayer hydrogel system with excellent self-healing and detachment properties irradiated by near-infrared irradiation was fabricated via a two-step in situ free radical polymerization. After the investigations of the effect of graphene-oxide content on the self-healing degree and detachment rate, a type of PDMAA hydrogel with a fast self-healing degree and a type of PNIPAm hydrogel with a rapid detachment rate were chosen as the components of a bilayer hydrogel system. The bilayer hydrogel can achieve specific functional applications by the structural design, which can be treated as candidates for medical dressing materials and soft actuators and robot materials.

## 2. Materials and Methods

### 2.1. Material

Monomer *N*,*N*-dimethylacrylamide (DMAA, stabilized with 500 ppm hydroquinone monomethyl ether) was purchased from KOHJIN Film & Chemicals Co., Ltd., Tokyo, Japan. Monomer *N*-isopropylacrylamide (NIPAAm, C_6_H_11_NO, Aladdin, Shanghai, China, 2% stabilizer) was recrystallized from toluene/*n*-hexane mixture and dried in vacuum at room temperature for 48 h. Nano-sized synthetic hectorite clay, Laponite XLS, (92.32 wt % of Mg_5.34_Li_0.66_Si_8_O_20_(OH)_4_Na_0.66_ and 7.68 wt % of Na_4_P_2_O_7_) was purchased from Rockwood, Ltd. (Moosburg, Germany) and used after being dried at 125 °C for 2 h. Initiator potassium peroxydisulfate (KPS, K_2_S_2_O_8_, Shanghai Aibi Chemical Reagent Co., Ltd., Shanghai, China, Analytical reagent AR), catalyst *N*,*N*,*N’*,*N’*-tetramethylethylenediamine (TEMED, Tianjin Weiyi Chemical Technology Co., Ltd., Tianjin, China, 98%), graphene oxide (GO, Suzhou Hengqiu Graphene Technology Co., Ltd., Suzhou, China, 95%), nanofibrillated cellulose (NFC, Guilin Qihong Technology Co., Ltd., Guilin, China, 1342 nm), α-cyclodextrin (Shanghai Yuanye biological Technology Co., Ltd., Shanghai, China), and methyl blue (Shanghai Aibi Chemical Reagent Co., Ltd., Analytical reagent AR) were used as received. Pure water was obtained by deionization and filtration with a Millipore purification apparatus (resistivity ≥18.2 MΩ·cm).

### 2.2. Synthesis of Bilayer Hydrogel

The bilayer hydrogel consisted of a self-healing layer and biological viscidity layer. The self-healing hydrogel layer was synthesized via in-situ free radical polymerization of DMAA in the nano-sized clay suspension with GO and NFC. Before the fabrication of the self-healing hydrogel, the pure water was degassed in the continuous nitrogen-saturated atmosphere for 2 h. The graphene oxide with different weights was first dispersed in 19.5 mL of pure water by ultrasonic radiation for 30 min and stirred for 30 min via a magnetic stirrer (Model DF-101S, Changchun Jiyu Technology Equipment Co., Ltd., Changchun, China). The XLS clay was added into the GO suspension, which was stirred for 1 h and ultrasonically radiated for 30 min. Then 20 mg of NFC was added and stirred for 1 h in an ice-water bath. The monomer DMAA was added into miscible liquids of GO, XLS, and NFC under a nitrogen-saturated atmosphere in an ice-water bath for another 2 h. Finally, 0.5 mL of KPS solution with a concentration of 40 mg/mL and 27 μL of TEMED catalyst was added under stirring. The solution was rapidly dumped into a laboratory-made rubber mold of 70 mm × 20 mm × 2 mm (length × width × thickness). The polymerization was conducted at 25 °C for 24 h to produce the self-healing hydrogel. The mole ratio of DMAA monomer, initiator, and catalyst in all suspensions was kept at 100:0.370:0.638. In order to investigate the effect of GO content on the self-healing property, 0, 1, 2, and 3 mg/mL GO was added in the hydrogel, respectively. In this paper, the self-healing hydrogels were defined as PDMAA-GO0, PDMAA-GO1, PDMAA-GO2, and PDMAA-GO3, where 0, 1 2, 3 represented the concentration of GO. In addition to the addition of 40 mg α-cyclodextrin after adding NFC, the other fabrication processes of biological viscidity hydrogel were similar to those of self-healing hydrogel. PNIPAm-GO0, PNIPAm-GO1, PNIPAm-GO2, and PNIPAm-GO3 were defined to represent concentrations of GO in biological viscidity hydrogels, respectively. The compositions of the self-healing and biological viscidity hydrogels are listed in Table 1 and Table 2, respectively.

**Figure 1:**
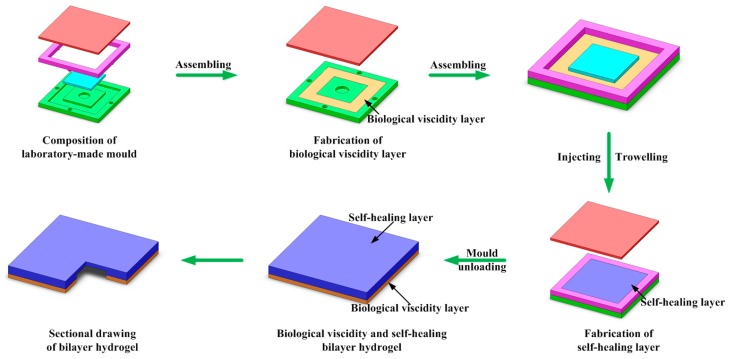
Schematic of the mold and fabrication process of bilayer hydrogel systems.

In order to study the conglutination, self-healing and detachment properties of bilayer hydrogel system, the hydrogel system with thickness of 2 mm were fabricated. The fabrication process which divided into two-step in situ free radical polymerization can be found in Figure 1. The biological viscidity layer with corresponding GO content was laid first in the laboratory-made mold. After the combination of watchet blue and light red molds, an interspace was formed. Then the PDMAA hydrogel was injected into the interspace on the biological viscidity layer and trowelled to obtain a smooth surface. After covered with scarlet coverplate, the bilayer hydrogel system was sealed in the laboratory-made mold. The polymerization of bilayer hydrogel system was also carried out at 25 °C for 24 h.

### 2.3. Characteristics

#### 2.3.1. Microstructure

In order to observe the internal microstructure via a scanning electron microscope (SEM) (Model Evo18 Carl Zeiss, Oberkochen, Germany) and an environmental scanning electron microscope (ESEM-FEG) (Model XL-30, FEI Company, Oregon, OR, USA), the corresponding samples were placed in liquid nitrogen. After freeze-drying in a freeze drying oven (LGJ-10C, Beijing Four Ring Scientific Instrument Factory Co., Ltd., Beijing, China) to remove water thoroughly, the hydrogels were sputtered with gold and observed.

#### 2.3.2. Infrared Spectrum and Differential Scanning Calorimetry (DSC) Analysis

Fourier transform infrared (FT-IR) spectra of PDMAA-GO and PNIPAm-GO hydrogels were recorded on IRAffinity-1 FT-IR spectrometer (Shimadzu Corporation, Kyoto, Japan). The range of wavenumber was 500–4000 cm^−1^. Before measurement, the samples were ground into powder and pressed with KBr into a disc.

The volume phase transition temperature (VPTT) of hydrogel sample was measured via differential scanning calorimetry (DSC) (Model Q2000, TA Company, Pennsylvania, PA, USA). The samples were heated from 20 to 60 °C, and then cooled from 60 to 20 °C at the rate of 10 °C/min under the nitrogen atmosphere. The VPTT of the hydrogel was determined at the onset of the endotherm peak during second heating.

#### 2.3.3. Photothermal Energy Transformation Efficiency

In order to measure the photothermal energy transformation efficiency of PDMAA hydrogels with different GO content, the PDMAA hydrogel were cut into blocks with dimensions of 10 mm × 10 mm × 2 mm (length × width × thickness) and irradiated by a near infrared illuminant with wavelength of 808 nm and power of 1.25 W (Model FC-W-808-30W, Changchun New Industries Optoelectronics Technology Co., Ltd., Changchun, China). The distance between light source and sample was 50 cm. The energy density delivered to the sample was 1.59 W/cm^2^. The temperature variation was measured by a thermocouple (Model PT100, Zhejiang Sanxing Thermometer Co., Ltd., Dongyang, China).

#### 2.3.4. Self-Healing Degree of PDMAA-GO Hydrogel

The PDMAA hydrogels used for self-healing test were cut into lath shape with dimension of 60 mm × 6 mm × 2 mm (length × width × thickness). The self-healing of snipped hydrogels was carried out by keeping the snipped surfaces in contact and irradiated with a near infrared illuminant with wavelength of 808 nm and power of 1.25 W for 1, 2, 3, 4, and 5 min in air, respectively. The distance between the light source and the sample was 50 cm. The energy density delivered to the sample was 1.59 W/cm^2^. The self-healing degree was defined as the tensile strength ratio of the healed PDMAA hydrogel to the original one. To obtain the tensile stress-strain characteristic of PDMAA and PNIPAm hydrogels, a universal testing machine (Model C43, MTS Criterion, Eden Prairie, MN, USA) with the constant loading rate of 100 mm/min was employed to test the tensile property. The stress was calculated via the ratio between the tensile load and cross-sectional area, and the strain was taken as the length change related to the original length. Average values of stress and strain were calculated from three individual measurements.

#### 2.3.5. Detachment Rate of PNIPAm Hydrogel

To obtain the detachment rate of PNIPAm hydrogels with various GO contents, the corresponding hydrogels sample with dimension of 70 mm × 20 mm × 2 mm (length × width × thickness) were attached on a white foam block by its biological viscidity directly. A piece of pigskin with dimensions of 20 mm × 20 mm × 2 mm (length × width × thickness) was attached on the hydrogel. A near infrared illuminant with wavelength of 808 nm and power of 1.25 W was used to irradiate the PNIPAm hydrogel. The distance between the light source and the sample was 50 cm. The energy density delivered to the sample was 1.59 W/cm^2^. Detachment rate was obtained by the time quantum from initiation of illumination to the drop of pigskin. A digital camera was used to record the whole process. Average values of the detachment rate were calculated from three individual measurements.

### 2.4. Functionalizational Application of Bilayer Hydrogel System

The PNIPAm layer of bilayer hydrogel system was attached on the pigskin via its biological viscidity. The methyl blue solution in a 1 mL injector was injected in the interspace between pigskin and PDMAA layer. After the remove of injector, a near infrared illuminant with wavelength of 808 nm and power of 1.25 W was used to irradiate the PDMAA layer to repair the pinhole. A digital camera was used to record the whole process.

## 3. Results and Discussion

### 3.1. Microstructure of the PDMAA and PNIPAm Hydrogel

**Figure 2:**
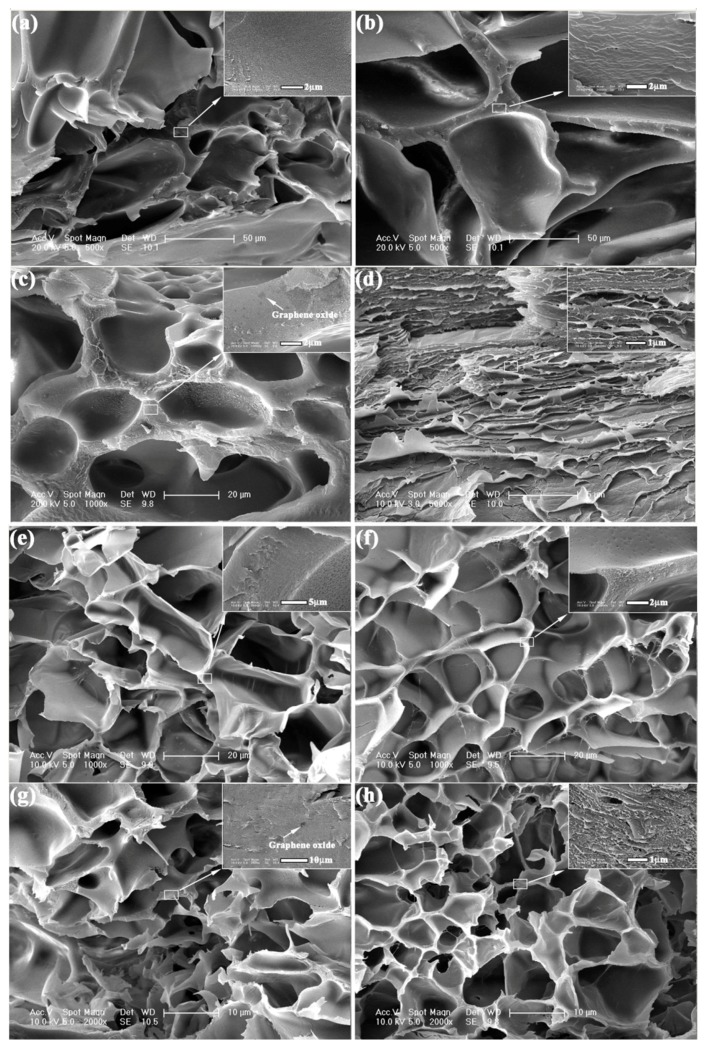
Microstructure of PDMAA hydrogels with various GO contents (**a**) 0 mg/mL; (**b**) 1 mg/mL; (**c**) 2 mg/mL; and (**d**) 3 mg/mL and PNIPAm hydrogels with various GO contents; (**e**) 0 mg/mL; (**f**) 1 mg/mL; (**g**) 2 mg/mL; and (**h**) 3 mg/mL.

In order to obtain the microstructure of the PDMAA and PNIPAm hydrogels with 0, 1, 2 and 3 mg/mL GO, respectively, the hydrogels were observed via ESEM-FEG. The related micrographs are shown in Figure 2a–h. It can be obviously found that the PDMAA and PNIPAm hydrogels presented a honeycomb-like structure and uniform distribution. The PDMAA hydrogel with 3 mg/mL GO content exhibited relative dense microstructure. Even though the monomer in Figure 2a–d was different from Figure 2e–h, the pore size became smaller along with the increase of GO content, indicating the high cross-linking density in the hydrogel network. Via the high magnification in the upper-right corner of Figure 2, the existence of microholes and graphene oxide can be found, which confirmed the effect of GO content on the microstructure characteristics of PDMAA and PNIPAm hydrogels.

### 3.2. FT-IR Spectra and DSC Analysis

**Figure 3:**
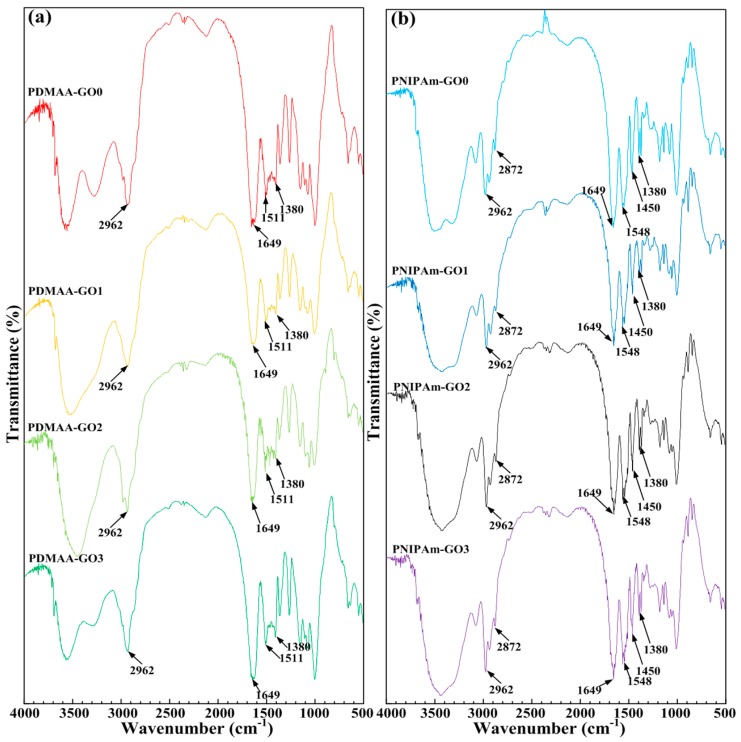
Typical FT-IR spectra characteristics of (**a**) PDMAA hydrogels and (**b**) PNIPAm hydrogels with various GO contents.

In order to ensure whether the addition of NFC and GO influence the existence of acylamino and isopropyl in a bilayer hydrogel or not, infrared spectrum experiments were conducted. The typical FT-IR spectra of the PDMAA and PNIPAm hydrogels with different GO contents are presented in Figure 3a,b, respectively. Variation of GO content exhibited relative tiny influence on the FT-IR profiles of the two kinds of hydrogels. As shown in Figure 3a, the bands of 2962 and 1380 cm^−1^ were the peak of –CH3. The peaks of 1649 and 1511 cm^−1^ were the C=O stretching vibration peak and N–H bending vibration peak, respectively, which proved the existence of acylamino of PDMAA hydrogel. In Figure 3b, 2962, 2872, 1450, and 1380 cm^−1^ were the peaks of –CH3. Compared with 1380 cm^−1^ in Figure 3a, the peak of 1380 cm^−1^ in Figure 3b was divided into two peaks, exhibiting the characteristic of isopropyl. The C=O stretching vibration peak of 1649 cm^−1^ and N–H bending vibration peak of 1545 cm^−1^ showed the characteristic peaks of amide. Namely, Figure 3b disclosed the appearance of hydrophilic acylamino and the hydrophobic isopropyl of *N*-isopropylacrylamide, which built the functional base of the thermoresponsive characteristic. Moreover, the functional chemical bonds of PDMAA hydrogels and PNIPAm hydrogels were not influenced by the addition of NFC and GO.

**Figure 4:**
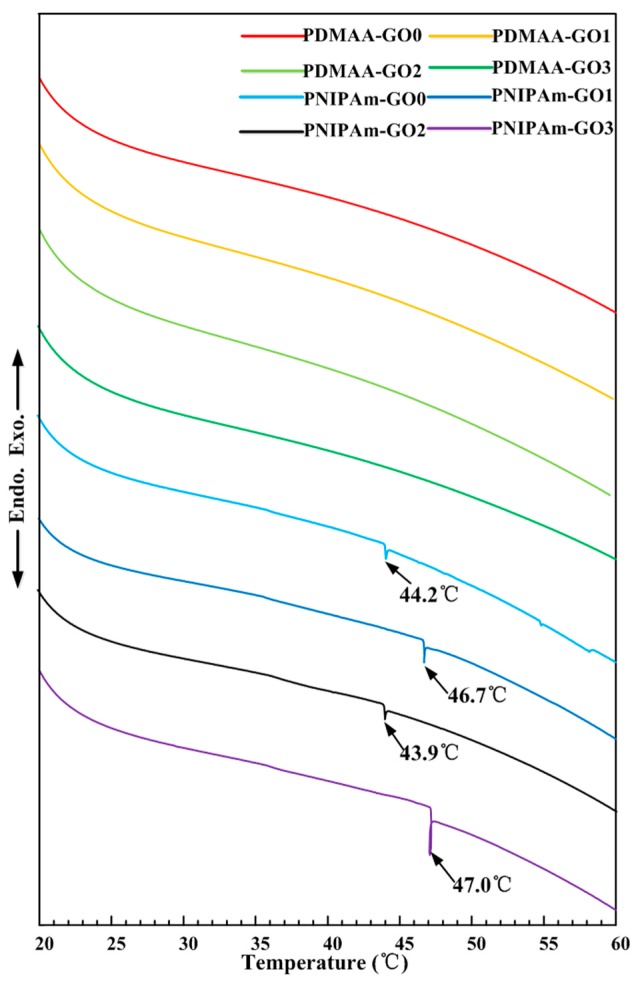
Differential Scanning Calorimetry (DSC) profiles of PDMAA and PNIPAm hydrogels with various GO contents.

Figure 4 shows the DSC profiles for the PDMAA and PNIPAm hydrogels with various GO contents. DSC profiles of PDMAA hydrogels indicated the insensitivity to temperature. On the contrary, PNIPAm hydrogels exhibited an obvious thermoresponsive characteristic. The volume phase transition temperature was range from 44.2 to 47 °C. In the view of functionalization application, without volume phase transition, GO in the PDMAA hydrogel absorbed near-infrared laser radiation and increased the temperature to promote the self-healing of the damaged part of the PDMAA hydrogel. Due to the existence of the volume phase transition, the PNIPAm hydrogel absorbed near-infrared laser radiation and increased the temperature to realize the detachment property. The insensitivity to temperature of the PDMAA hydrogel and the sensitivity to temperature of the PNIPAm hydrogel provided the feasibility of a combination of a bilayer hydrogel system. Therefore, in order to investigate the effect of GO contents on the self-healing property of the PDMAA hydrogel and the detachment rate of the PNIPAm hydrogel, experiments of the photothermal energy transformation efficiency, self-healing degree, and detachment rate were conducted.

### 3.3. Self-Healing Property

#### 3.3.1. Photothermal Energy Transformation Efficiency

**Figure 5:**
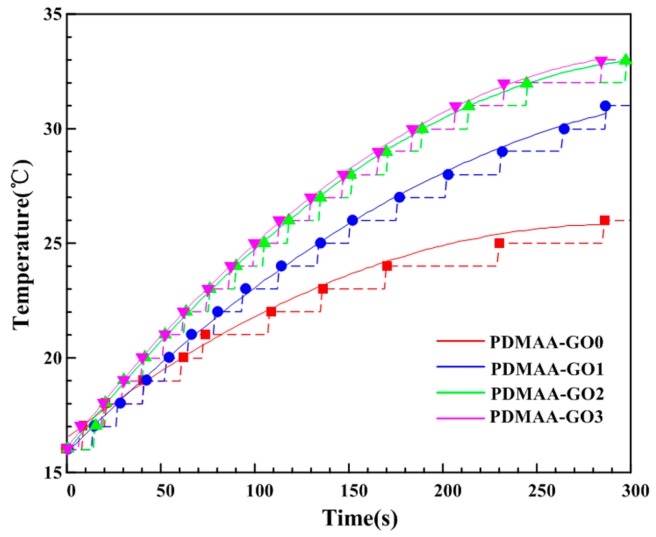
Photothermal energy transformation efficiency of PDMAA hydrogels with various GO contents.

The results of photothermal energy transformation efficiency of PDMAA hydrogels with various GO contents are shown in Figure 5. The dotted lines in Figure 5 represent the real-time record of the temperature variation. The solid lines in Figure 5 represent the fitting of the corresponding dotted lines to exhibit photothermal energy transformation efficiency. From Figure 5 it can be observed that, with the increase of GO content, the heating rate of the hydrogels increased. The heating rate of PDMAA-GO3 was similar to PDMAA-GO2 but, over time, the time for reaching a similar temperature of PDMAA-GO3 was earlier than that of PDMAA-GO2. Namely, the PDMAA hydrogel with a relative high amount of GO content exhibited a relative high photothermal energy transformation efficiency, which was beneficial for the rapid self-healing degree.

#### 3.3.2. Self-Healing Degree

**Figure 6:**
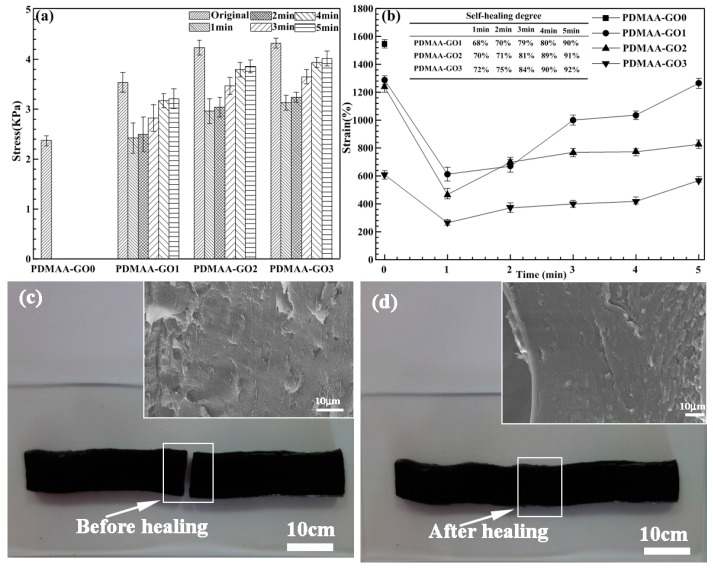
(**a**) Stress values, (**b**) strain values, and the corresponding self-healing degree of PDMAA hydrogels with different GO contents and self-healing time, morphology, and microstructure of PDMAA-GO3 hydrogel (**c**) before and (**d**) after 5 min of self-healing.

Figure 6 shows the self-healing degree of PDMAA hydrogels with different GO contents and self-healing time, and the corresponding microstructure of PDMAA-GO3 before and after self-healing. In order to compare the self-healing degree of hydrogel samples, the original stress values were tested first. The self-healing degree was defined as the stress ratio of the self-healed sample to the corresponding one. Due to the insensitivity to photothermal energy transformation, self-healing experiments of PDMAA-GO0 were not conducted. The addition and variation of GO content excellently affected the corresponding mechanical strength and strain. PDMAA hydrogels with various GO contents exhibited higher stress values and lesser strain values than those of PDMAA-GO0, respectively. With the increase of GO content, the stress and strain values of PDMAA-GO1, PDMAA-GO2, and PDMAA-GO3 increased and decreased, respectively. The hydrogel with 3 mg/mL GO content presented the highest strength and lowest strain, as shown in Figure 6a. Under the irradiation of near infrared laser for 1, 2, 3, 4, and 5 min, respectively, PDMAA hydrogels with 1, 2, and 3 mg/mL GO presented different self-healing degrees, as shown in Figure 6b. The self-healing degree of PDMAA hydrogels increased along with the increase of the irradiation time. Under the condition of the same amount of time, the PDMAA hydrogel with higher GO content exhibited a higher self-healing degree. After 5 min, the maximal self-healing degree of PDMAA-GO3 achieved 92%, indicating an excellent self-healing property. Figure 6c,d shows the morphology and microstructure of the PDMAA-GO3 hydrogel before and after 5 min of self-healing. Attributed to the absence of complete swelling, the hydrogel exhibited relatively higher density than that of Figure 2d. Before near-infrared irradiation, the hydrogel presented a smooth fracture surface. After 5 min of self-healing, besides small cracks, the healed hydrogel showed a very dense microstructure, which was beneficial for the enhancement of the self-healing degree.

Combined with Figure 2, the increasing GO content enhanced the cross-linking density and compactness, leading to the high mechanical strength and low strain. The photothermal energy transformation property of GO was the key point for the self-healing behavior. After the fracture surfaces of the cut PDMAA hydrogel being contacted with each other, attributed to the mutual diffusion phenomenon, the polymer chains of PDMAA moved across the fracture surfaces to the opposite side [10]. The diffused PDMAA polymer chains built hydrogen bonds with XLS clay and GO. With the increase of the amount of hydrogen bonds, the network structure of PDMAA hydrogel was rebuilt, and the damaged fracture surfaces were self-healed. The higher temperature promoted the mutual diffusion of hydrogen bonds. Combined with Figure 5, the existence of GO transformed the near-infrared laser radiation into heat. Increasing the GO content resulted in the increase of the heating rate, which promoted the self-healing degree, as shown in Figure 6.

### 3.4. Detachment Property Analysis

#### 3.4.1. Mechanical Properties of PNIPAm Hydrogel

**Figure 7:**
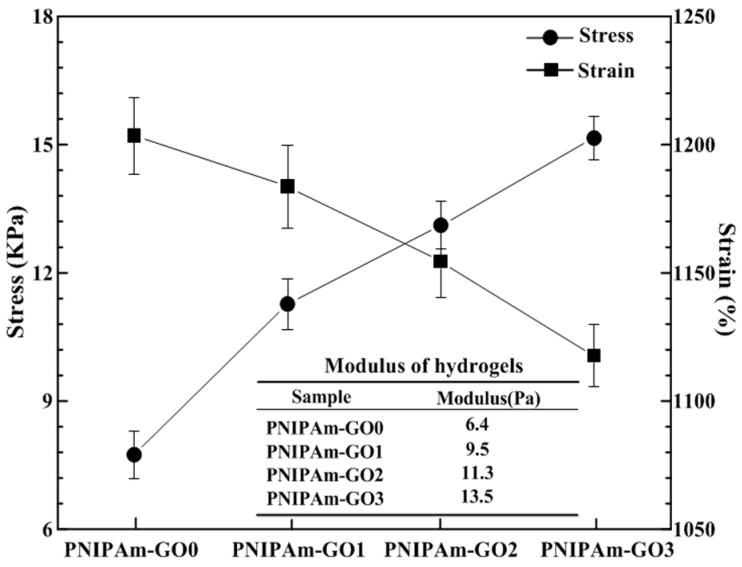
Stress, strain, and modulus values of PNIPAm hydrogels with various GO contents.

After the investigation of the self-healing degree of PDMAA hydrogels, PDMAA-GO3 exhibited the highest mechanical strength and self-healing degree. As the other layer of the bilayer hydrogel system, besides biological viscidity and detachment properties, PNIPAm hydrogel should have high mechanical strength. Therefore, the tensile experiment of PNIPAm hydrogels with different GO contents was conducted, as shown in Figure 7.

The variation of GO contents played an important role in the mechanical properties of the PNIPAm hydrogels. With the increase of GO content, the stress and strain values increased and decreased, respectively. The stress values of PNIPAm-GO0, PNIPAm-GO1, PNIPAm-GO2, and PNIPAm-GO3 hydrogels are 7.7, 11.3, 13.1 and 15.1 KPa, respectively. The corresponding strain values are 1203.4%, 1183.5%, 1154.4%, and 1117.6%, respectively. Moreover, the modulus increased from 6.4 to 13.5 Pa. The PNIPAm hydrogel with 3 mg/mL GO content presented the highest mechanical strength. Combined with Figure 2, the cross-linking density of PNIPAm hydrogels enhanced along with the increase of GO contents, leading to high strength and low strain.

#### 3.4.2. Detachment Rate of PNIPAm Hydrogel

The existence of α-cyclodextrin promoted the biological viscidity of PNIPAm hydrogels. In practical applications, how to strip the PNIPAm hydrogel with biological viscidity rapidly and integrally via irradiation of a near-infrared laser was a key point for the application of the bilayer hydrogel system. Therefore, the corresponding detachment rate experiments were conducted.

**Figure 8:**
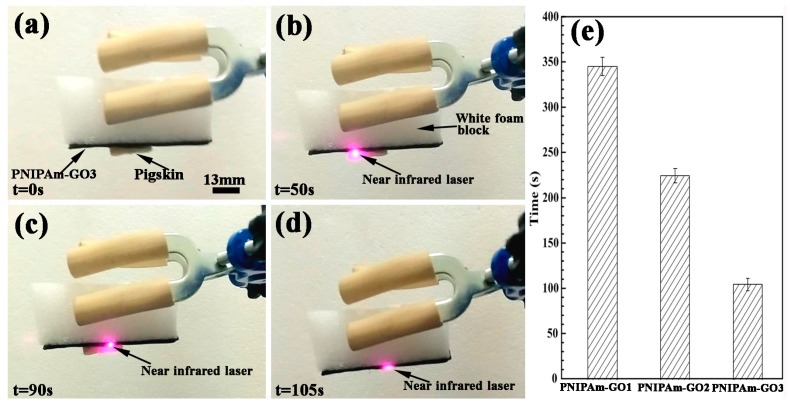
(**a**–**d**) detachment process of PNIPAm-GO3 hydrogel and (**e**) the detachment rate of PNIPAm hydrogels with 1, 2, and 3 mg/mL GO.

Figure 8 shows the detachment process of the PNIPAm-GO3 hydrogel and the detachment rate of PNIPAm hydrogels with 1, 2, and 3 mg/mL GO. In the initial state, the pigskin was attached to the surface of the PNIPAm-GO3 hydrogel, as shown in Figure 8a. The time of 0 s was defined as the time of irradiation of the near-infrared laser. In order to realize the detachment of pigskin, the near-infrared laser was repeatedly irradiated on the flank surface of the hydrogel in contact with the pigskin, as shown in Figure 8b,c. When the time reached 105 s, the pigskin was stripped, realizing successful detachment, as shown in Figure 8d. The PNIPAm hydrogels with 1 and 2 mg/mL GO contents exhibited similar detachment processes of PNIPAm-GO3 hydrogel. However, the variation of GO content influenced the corresponding detachment rate, which can be seen in Figure 8e. The average time of PNIPAm hydrogels with 1, 2, and 3 mg/mL GO contents for the detachment process were 345, 225, and 105 s, respectively. Namely, with the increase of GO content, the detachment rate of PNIPAm hydrogels increased.

Combined with Figure 5, GO exhibited a high photothermal energy transformation property. Due to the thermoresponsive characteristic, when the temperature of PNIPAm hydrogel was higher than the volume phase transition temperature in Figure 4, the water in the PNIPAm hydrogel appeared at the interface between the hydrogel and pigskin, which reduced the corresponding biological viscidity and stripped pigskin. The high amount of GO content promoted the photothermal energy transformation efficiency and the speed for obtaining the volume phase transition temperature, which increased the detachment rate, as shown in Figure 8e.

Combined with Figure 6 and Figure 8, the PDMAA-GO3 hydrogel exhibited the highest mechanical strength and self-healing degree. The PNIPAm-GO3 hydrogel showed the highest mechanical strength, biological viscidity, and detachment rate. Therefore, the characteristics of PDMAA-GO3 and PNIPAm-GO3 hydrogels provided the functional base for the feasibility of fabrication and application of a bilayer hydrogel system with rapid self-healing, biological viscidity, and detachment rate properties. In order to fabricate the bilayer hydrogel system, PDMAA-GO3 and PNIPAm-GO3 hydrogels were chosen as component materials via a two-step in situ free radical polymerization in the laboratory-made mold in Figure 1 to realize the summary practical application.

### 3.5. Microstructure and Application of the Bilayer Hydrogel System

#### 3.5.1. Microstructure Characteristics

**Figure 9:**
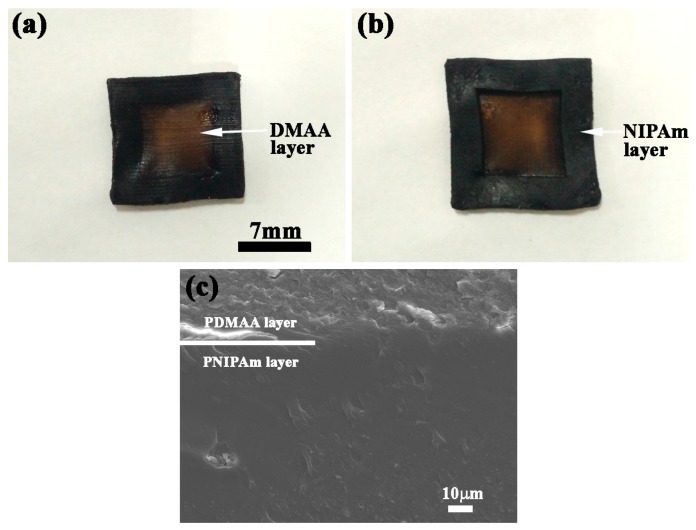
Morphology of (**a**) PDMAA layer; (**b**) PNIPAm layer; and (**c**) the interface bonding characteristics of bilayer hydrogel.

Figure 9a,b shows the intuitionistic stratification morphology of the bilayer hydrogel system with self-healing, biological viscidity, and detachment properties, which consists of PDMAA-GO3 and PNIPAm-GO3 hydrogels. The PDMAA layer and the PNIPAm layer exhibited excellent shape and structure, which proved the functionality of the molds and fabrication methods. Figure 9c shows the SEM micrographs of interface bonding characteristics between the PDMAA layer and the PNIPAm layer. The bilayer hydrogel exhibits high bonding strength, and the clear boundary has been marked. Due to the plenitudinous crosslinks across the interface, the two layers were locked together tightly. The firm bilayer structure built a structure base for the application of the bilayer hydrogel system driven by near infrared laser stimulation. In order to investigate the feasibility of the functional application, the corresponding adherency and self-healing experiments were conducted.

#### 3.5.2. The Application of the Bilayer Hydrogel System

**Figure 10:**
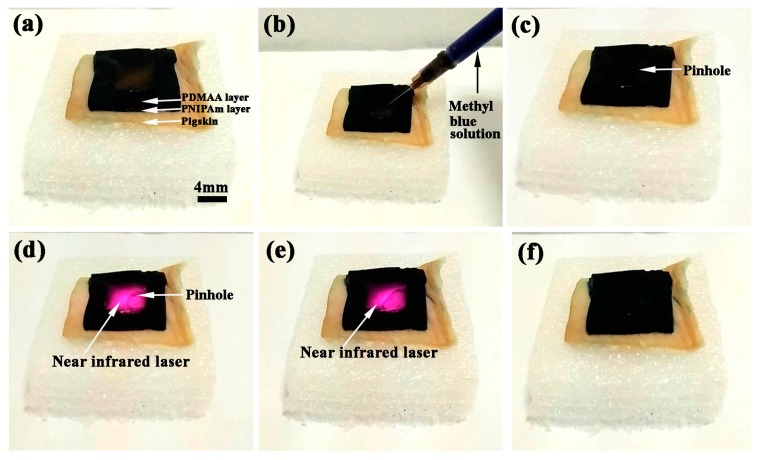
The adherency and self-healing process of bilayer hydrogel (**a**) covered on the pigskin; (**b**) and (**c**) injection of methyl blue solution; (**d**,**e**) self-healing process of pinhole irradiated by near-infrared irradiation for 115 s; and (**f**) morphology of bilayer hydrogel after self-healing.

In order to demonstrate the summary practical application of the bilayer hydrogel in the medical field, such as a self-healing medical dressing material with biological viscidity and detachment property, the bilayer hydrogel system was covered on a piece of pigskin, as shown in Figure 10a. Attributed to the structure design of Figure 1, the interspace formed between the PDMAA layer and the pigskin was used for reserving liquid medicine for wound healing. Here, the methyl blue solution was used for the replacement of the liquid medicine. As shown in Figure 10b, the methyl blue solution was injected into the interspace via impaling the PDMAA layer. After injection, a pinhole stayed on the PDMAA surface, which can be found in Figure 10d. Combined with the self-healing property of PDMAA-GO3 hydrogel, the pinhole on PDMAA layer can be repaired by near-infrared laser. Therefore, a near-infrared laser with 1.25 W irradiated the place of the pinhole, as shown in Figure 10d. The time of the initiation of the irradiation of the near-infrared laser was defined as 0 s. To ensure the complete self-healing degree of the pinhole, the near infrared laser was removed at 115 s. After 115 s, the pinhole was completely repaired. From Figure 10e, no existence of the pinhole can be found. Figure 10f shows the final state of the bilayer hydrogel after self-healing. The injected methyl blue solution was sealed in the interspace. Under the irradiation by the near-infrared laser, the bilayer hydrogel system can be stripped. The fabricated bilayer hydrogel system presented the combination property of PDMAA-GO3 and PNIPAm-GO3 hydrogels and exhibited the biological viscidity, self-healing, and detachment properties. Due to the characteristics of easy fabrication and low cost, the bilayer hydrogel system can be widely used in the medical field and in soft robots which need self-healing properties. 

## 4. Conclusions

In this paper, a novel type of bilayer hydrogel system with excellent self-healing and detachment properties, achieved by near-infrared irradiation, was successively fabricated via a two-step in situ free radical polymerization. As the components of bilayer hydrogel system, the cross-linking density, resulting from a variation of GO content, led to high mechanical strength of PDMAA layer and PNIPAm layer. Due to the high photothermal energy transformation property of GO, photothermal energy transformation efficiency of PDMAA hydrogels enhanced with the increase of GO content, which significantly affected the corresponding self-healing rate. After 5 min near-infrared irradiation, the PDMAA-GO3 hydrogel reached strength recovery of 92%. The existence of α-cyclodextrin promoted the biological viscidity of PNIPAm hydrogels. The addition of GO built the functional base for the detachment of PNIPAm hydrogel from pigskin. The detachment rate increased along with the increase of GO content. Attributed to the plenitudinous crosslinks across the interface, PDMAA-GO3 layer and PNIPAm-GO3 layer were locked together tightly, providing the structural base for the practical application. Via the effective mold design, a potential application of the bilayer hydrogel system was treated as a medical dressing material with viscidity, self-healing, and detachment, which can reserve the injected medicine between the bilayer hydrogel system and skin. After curing, under the irradiation of a near-infrared laser, the bilayer hydrogel system can be stripped. This bilayer hydrogel system can also be applied in the medical field, in soft robots, and so on.

## Figures and Tables

**Table 1:** The compositions of the poly *N*,*N*-dimethylacrylamide (PDMAA) hydrogels.

Sample	DMAA (g)	GO (mg)	NFC (mg)	XLS (g)	KPS (mg)	TEMED (μL)	H_2_O (mL)
PDMAA-GO0	1.98	0	20	0.682	20	27	20
PDMAA-GO1	1.98	20	20	0.682	20	27	20
PDMAA-GO2	1.98	40	20	0.682	20	27	20
PDMAA-GO3	1.98	60	20	0.682	20	27	20

**Table 2:** The compositions of the poly *N*-isopropylacrylamide (PNIPAm) hydrogels.

Sample	NIPAm (g)	GO (mg)	α-Cyclodextrin (mg)	NFC (mg)	XLS (g)	KPS (mg)	TEMED (μL)	H_2_O (mL)
PNIPAm-GO0	2.26	0	40	20	0.692	20	27	20
PNIPAm-GO1	2.26	20	40	20	0.692	20	27	20
PNIPAm-GO2	2.26	40	40	20	0.692	20	27	20
PNIPAm-GO3	2.26	60	40	20	0.692	20	27	20
